# Evaluating Validation Strategies in Motor Imagery EEG: A Full-Cohort GAF–PLV Analysis and Matched Sensitivity Study

**DOI:** 10.3390/brainsci16080873

**Published:** 2026-08-17

**Authors:** Wenwen Chang, Hesam Akbari, Muhammad Tariq Sadiq, Renjie Lv, Rab Nawaz

**Affiliations:** 1 School of Electronic and Information Engineering, Lanzhou Jiaotong University, Lanzhou 730070, China; changww2013@126.com (W.C.); lvrenjie2022@163.com (R.L.); 2 Department of Information Science, University of North Texas, Denton, TX 76207, USA; hesam.akbari@unt.edu; 3 School of Computer Science and Electronic Engineering, University of Essex, Wivenhoe Park, Colchester CO4 3SQ, UK; rnawaz@essex.ac.uk

**Keywords:** motor imagery EEG, validation protocol, cross-subject generalisation, subject-independent evaluation, leave-one-subject-out cross-validation, nested cross-validation, data leakage

## Abstract

**Background:** Performance estimates in motor-imagery electroencephalography (MI-EEG) can depend strongly on how observations are partitioned for training, model selection, and testing. Random sample- or window-level splitting may place data from the same participant in different folds and therefore does not answer the same question as evaluation on previously unseen participants. **Methods:** We evaluated a previously developed Gramian angular field–phase-locking value (GAF–PLV) classifier on the retained full cohort (N=105) using binary left-versus-right MI and leave-one-subject-out cross-validation (LOSO). Separately, a predefined, outcome-independent subset (N=30) was used for a matched sensitivity analysis of eight classifiers, binary and four-class tasks, and three validation strategies: random five-fold cross-validation, LOSO, and nested LOSO with subject-grouped inner model selection. **Results:** In the full-cohort GAF–PLV analysis, mean accuracy was 58.07% ± 8.27% and Macro-F1 was 53.48% ± 11.19%, with substantial between-subject variability. In the predefined matched subset, performance estimates and numerical model rankings changed across validation strategies. For example, the numerically highest binary-accuracy model was ShallowConvNet under random five-fold cross-validation, DeepConvNet under LOSO, and ATCNet under nested LOSO. **Conclusions:** Random within-cohort classification and generalisation to previously unseen subjects are distinct evaluation targets. MI-EEG reports should state the cohort, partition unit, validation design, and model-selection procedure. Rankings in the multi-model analysis are conditional on the predefined 30-subject subset and common 0.4 s input setting and are not presented as definitive full-cohort or architecture-optimal rankings.

## 1. Introduction

Brain–computer interfaces (BCIs) can be built from several types of neural measurements. Non-invasive examples include scalp electroencephalography (EEG), magnetoencephalography (MEG), and haemodynamic approaches such as functional near-infrared spectroscopy (fNIRS), whereas invasive systems can use electrocorticographic or intracortical signals [1,2,3,4]. The present work concerns scalp EEG because it is non-invasive, comparatively portable, and has high temporal resolution [5,6]. Motor imagery (MI) is widely studied in EEG-based BCI research for assistive control and rehabilitation-oriented applications, but clinical translation should be distinguished from routine clinical care: controlled studies have demonstrated the potential of BCI-assisted neurorehabilitation, while such studies do not by themselves establish a standard-of-care technology [7].

MI-EEG decoding remains difficult because recordings are non-stationary and show substantial inter-individual variability. Classical pipelines use spatial or spectral features followed by classifiers such as support-vector machines, whereas deep-learning approaches include compact convolutional networks, deeper convolutional models, attention mechanisms, transformers, graph models, and transformed representations [8,9,10,11,12,13]. These model families make different assumptions about temporal receptive fields, spatial structure, and regularisation, so an apparently simple comparison of headline accuracies can confound architecture with the evaluation design.

The partition unit is especially consequential when multiple windows or trials are derived from each participant. If samples from the same participant occur in both training and test folds, the model can exploit subject-specific structure that would not be available for a previously unseen participant. Direct EEG investigations have shown that segment-wise splitting can overestimate performance relative to subject-wise holdout, and recent MI-EEG work has likewise emphasised the role of data partitioning in cross-subject evaluation [14,15,16]. This does not make random sample-level cross-validation intrinsically invalid: it estimates within-cohort classification under that partitioning scheme. It does, however, make that estimate scientifically different from generalisation to unseen subjects.

The PhysioNet EEG Motor Movement/Imagery dataset is a useful setting in which to study this distinction because it is large, public, and has been analysed under heterogeneous cohort and partitioning choices [17,18]. Published work has used pooled or sample-level evaluation, subject-oriented evaluation, and reduced-cohort benchmarking for different objectives [19,20,21,22,23,24,25,26]. The existence of reduced-cohort studies does not establish that a smaller cohort is statistically representative of the full dataset; it only shows that reduced-cohort benchmarking is an established experimental practice whose inferential scope must be stated explicitly. Representative cohort choices are therefore summarised in Supplementary Table S1 rather than in the main text.

The objective of this study is to determine how validation strategy changes MI-EEG performance estimates and numerical model rankings while maintaining a clear distinction between two analytical levels. The primary analysis evaluates the previously developed GAF–PLV framework [13] on all 105 retained subjects for binary left-versus-right MI using LOSO. A separate, predefined 30-subject sensitivity analysis evaluates eight classifiers on binary and four-class tasks under random five-fold cross-validation, LOSO, and nested LOSO. We hypothesised that (i) random sample-level partitions would generally yield more optimistic estimates than subject-wise evaluation and (ii) numerical rankings would not be invariant to the validation strategy. The multi-model analysis is intentionally interpreted within its predefined subset and common input setting rather than as a definitive full-cohort classifier leaderboard.

## 2. Methodology

### 2.1. EEG Dataset Description and Problem Formulation

The experiments use the public PhysioNet EEG Motor Movement/Imagery Dataset, released by Schalk and colleagues and recorded with the BCI2000 system [17,27]. The release contains recordings from 109 participant directories, with 64 scalp EEG channels arranged according to the international 10–10 system and sampled at 160 Hz. Each participant completed 14 runs: two 1-min baselines (eyes open and eyes closed) and three repetitions of four 2-min motor execution/imagery conditions. In the imagery runs relevant here, event labels distinguish rest from left/right fist imagery or both-fists/both-feet imagery according to run type [17]. The classification epochs used in the submitted pipeline contain 4 s of MI activity, corresponding to 640 samples per channel.

The authoritative public release does not provide complete subject-level age, sex, and handedness information required for demographic or handedness-stratified analysis; consequently these characteristics cannot be retrospectively reported or analysed. We therefore avoid assigning undocumented demographic characteristics to the cohort.

**Figure 1:**
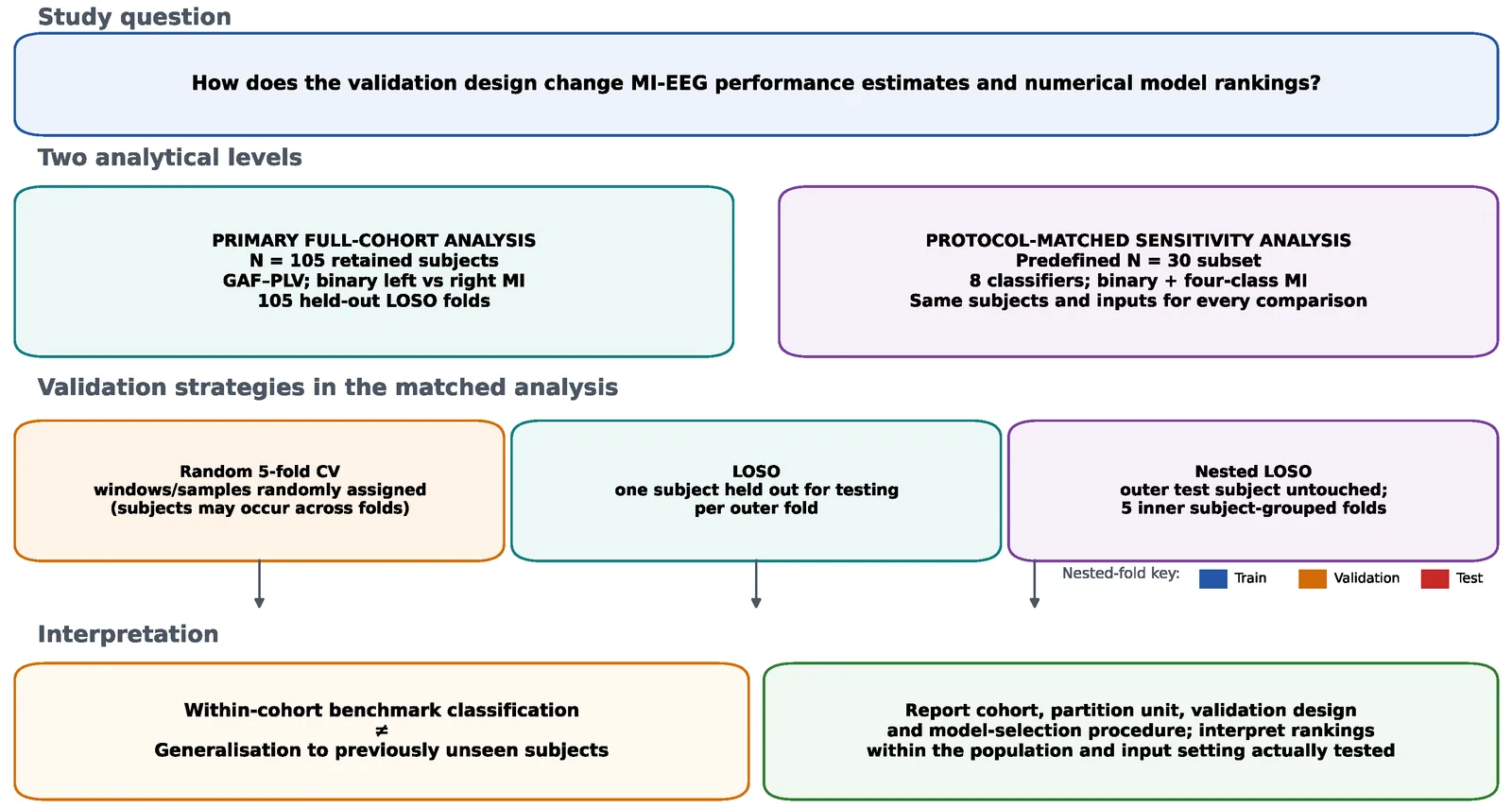
Overview of the study design and evaluation framework. The primary full-cohort analysis evaluates the GAF–PLV framework on the retained N=105 binary cohort under LOSO. The separate protocol-matched sensitivity analysis uses a predefined N=30 subset to compare eight classifiers and three validation strategies. The nested-fold colour key distinguishes training, inner validation, and held-out outer testing.

Figure 1 summarises the study design and makes the two analytical levels explicit.

The primary full-cohort GAF–PLV analysis is performed on the retained 105-subject binary left-versus-right MI task. The protocol-matched sensitivity analysis additionally considers binary and four-class MI tasks on a predefined 30-subject subset, as described in Section 2.5. Let the EEG trial from subject *s*, trial *r*, channel *c*, and time index *t* be denoted by

(1) $$x_{s,r,c}\left(t\right),\;s\in S,\;r\in R,\;c\in \left\{1,\ldots ,64\right\},\;t\in \left\{1,\ldots ,640\right\}.$$

For the binary task,

(2) $$y_{s,r}^{\left(2\right)}\in \left\{0,1\right\},$$

where class 0 denotes left-hand MI and class 1 denotes right-hand MI. For the four-class task,

(3) $$y_{s,r}^{\left(4\right)}\in \left\{0,1,2,3\right\},$$

corresponding to the four MI classes used in the protocol-matched comparative experiments.

### 2.2. Subject Inclusion and Reporting Transparency

The official dataset provides participant directories S001–S109 [17]. The retained binary GAF–PLV LOSO analysis available for this study comprises subject IDs 1–105; all 105 available held-out folds are included. We therefore define this as the primary full-cohort analysis for the retained cohort, rather than implying that results exist for excluded identifiers 106–109.

The protocol-matched sensitivity analysis uses a predefined 30-subject subset selected from this retained cohort, as described in Section 2.5. It answers a different question: whether performance estimates and numerical rankings change when validation strategy is changed under matched data conditions. It is not used to claim population or demographic representativeness of those 30 subjects.

### 2.3. Preprocessing and Temporal Partitioning

Each trial was filtered in the 8–30 Hz motor-rhythm band before feature construction. This band was selected because it covers the sensorimotor mu and beta rhythms commonly associated with motor imagery-related ERD/ERS [28,29]. Frequencies below 8 Hz were not separately analysed because the present decoding pipeline focuses on motor-rhythm activity rather than lower-frequency components that are more susceptible to slow drift, ocular activity, and non-motor-related fluctuations.

Common average reference was applied independently at each time point:

(4) $${\tilde{x}}_{s,r,c}\left(t\right)=x_{s,r,c}\left(t\right)-\frac{1}{C}\sum_{k=1}^{C}x_{s,r,k}\left(t\right),\;C=64.$$

Each channel was then z-scored independently within the trial:

(5) $$z_{s,r,c}\left(t\right)=\frac{{\tilde{x}}_{s,r,c}\left(t\right)-\mu_{s,r,c}}{\sigma_{s,r,c}+\varepsilon },$$

where

(6) $$\mu_{s,r,c}=\frac{1}{T}\sum_{t=1}^{T}{\tilde{x}}_{s,r,c}\left(t\right),$$

(7) $$\sigma_{s,r,c}=\sqrt{\frac{1}{T-1}\sum_{t=1}^{T}{\left({\tilde{x}}_{s,r,c}\left(t\right)-\mu_{s,r,c}\right)}^{2}}.$$

Here, T=640 and ε is a small numerical constant used to avoid division by zero.

Each 4 s trial was divided into ten non-overlapping windows of length 0.4 s. Since the sampling rate is 160 Hz, each window contains

(8) L=0.4×160=64

samples, and each trial contains

(9) $$W=\frac{640}{64}=10$$

windows. The *w*-th window is denoted by $Z_{s,r}^{\left(w\right)}\in R^{L\times C}$.

The 0.4 s non-overlapping window was retained because it is the temporal unit used by the previously developed GAF–PLV pipeline [13]. Using that same duration for every classifier in the sensitivity analysis keeps temporal exposure and the sample-partition unit fixed while the validation strategy changes. It should not be interpreted as a literature-derived universal optimum. Original evaluations of EEGNet, ATCNet, and EEG-Conformer used substantially longer task segments than 0.4 s [8,10,11]; consequently, the matched comparison characterises the implementations under the common 0.4 s setting, not each architecture under its architecture-specific optimal input duration. Model-specific pooling or patch settings were adjusted only where necessary to maintain valid temporal operations, as reported in Supplementary Table S3.

### 2.4. Primary GAF–PLV–CNN Framework and Compared Classifiers

The present study uses the previously developed GAF–PLV–CNN as the primary framework and includes representative classifiers as matched reference models. This design separates the full-cohort assessment of the primary framework from the multi-model sensitivity question.

For the primary GAF–PLV–CNN framework, each preprocessed EEG window is represented by two complementary descriptors. A Gramian angular field (GAF) encodes a one-dimensional time series in an image-like matrix while retaining ordered temporal relations [30]. A GAF matrix is computed for each EEG channel, and each channel-wise matrix is vectorised. Spearman rank correlation is then calculated between pairs of vectorised GAF descriptors, producing a 64 × 64 matrix of monotonic similarity between channel-specific temporal patterns. Spearman correlation was chosen as a nonparametric similarity summary rather than as a physical spatial-distance measure. There is no claim that this operation encodes scalp geometry.

In the second branch, phase-locking value (PLV) is computed between channel pairs from Hilbert-transform phase, producing a 64 × 64 functional phase-synchrony matrix [31]. PLV captures functional phase relationships; it is not equivalent to anatomical or geometric electrode topology. Thus, the present representation preserves channel identity and pairwise temporal/functional relationships but does not explicitly encode electrode coordinates, Euclidean or geodesic distances, or a neighbourhood graph. Geometry-aware graph or topographic modelling is a distinct design choice, as illustrated by EEG graph approaches [12].

Both descriptors are converted into CNN-compatible image inputs using the same deterministic procedure used in the previously developed framework [13]. Each 64 × 64 descriptor is mapped to an RGB pseudocolour image and resized to 256 × 256 pixels using bilinear interpolation. The colour channels therefore visualise the scalar descriptor through a fixed mapping; they do not constitute independent EEG channels or additional physiological variables. We retained this transformation for fidelity to the reference framework rather than claiming a specific physiological advantage of pseudocolour encoding.

The two image inputs are decoded by a dual-input parallel CNN. The temporal branch receives the GAF–Spearman image, and the spatial branch receives the PLV image. Branch-specific convolutional features are extracted separately and concatenated before final softmax classification. The fused representation can be written as

(10) $$h=[h^{\left(T\right)};h^{\left(S\right)}],$$

where $h^{\left(T\right)}$ and $h^{\left(S\right)}$ denote the temporal and spatial branch embeddings, respectively. The final prediction is obtained as

(11) $$\hat{y}=\mathrm{softmax}\left(Wh+b\right).$$

Training uses sparse categorical cross-entropy,

(12) $$L=-\sum_{k=1}^{K}⊮\left(y=k\right)\log{\hat{y}}_{k},$$

with the Adam optimiser, a maximum of 100 epochs, batch size 64 window representations, and early stopping during inner model-selection runs. Detailed layer-wise architecture and hyperparameter settings are provided in the Supplementary Material.

The protocol-matched comparison additionally includes CSP-SVM [28,32], FBCSP-SVM [33], EEGNet [8], DeepConvNet [9], ShallowConvNet [9], ATCNet [10], and EEG-Conformer [11]. These models were selected to cover classical machine learning, compact convolutional networks, and attention- or transformer-based deep learning architectures commonly used in MI-EEG classification.

### 2.5. Protocol-Matched Baseline Evaluation

All compared classifiers were evaluated using the same preprocessing pipeline, task definitions, performance metrics, and validation strategies. This matched design allows the effect of validation strategy to be examined while keeping the subjects, temporal input, preprocessing, task formulation, and evaluation metrics fixed across models.

For the matched comparison, a predefined subset of 30 subjects was selected from the retained 105-subject cohort. Before any model fitting, subject identifiers were divided into three ranges (1–35, 36–70, and 71–105), and a fixed random seed (2026) was used to sample 10 identifiers from each range. Subject selection was completed before model fitting and without access to classifier performance; therefore, no performance-based inclusion or exclusion criterion was used. The fixed seed and complete list of selected identifiers in Supplementary Table S2 make this outcome-independent procedure reproducible. The subject-ID ranges are not biological strata: this sampling only distributes the subset across the available index range and prevents concentration in a single contiguous block. It does not demonstrate demographic, physiological, or performance representativeness. The same 30 subjects were used for every classifier, task, and validation strategy, so comparisons within this predefined sensitivity analysis are matched.

Reduced-cohort PhysioNet MI-EEG benchmarking has precedent in published studies, including evaluations with 10, 20, 60, or 100 subjects by Lun et al., 10 or 20 subjects by Majoros and Oniga, 10 subjects by Hwaidi and Chen, 20 PhysioNet subjects by Fan et al., and 20 subjects by Aung et al. [22,23,24,25,26]. These examples demonstrate an established experimental practice but do not prove that any particular reduced subset is sufficient for full-cohort ranking stability. Accordingly, the present N=30 analysis is explicitly a sensitivity analysis, while stability of the eight-model rankings on all 105 retained subjects remains untested.

The exact workload also clarifies why the multi-model nested analysis was scoped separately. Supplementary Table S3 specifies three candidate configurations per model, five inner subject-grouped folds, and one final refit in every outer fold. For eight models and two tasks, the nested component at N=30 therefore entails 30 × 8 × 2 × [(5 × 3)+1]=7680 model fits/refits. Repeating the same design at N=105 would entail 105 × 8 × 2 × [(5 × 3)+1]=26,880 fits/refits, an exact 3.5-fold expansion of the nested component. This workload calculation supports the scope decision but is not used as evidence that the 30-subject rankings generalise to the full cohort.

### 2.6. Validation Protocols and Model Selection

Three validation protocols were used to examine how evaluation design affects MI-EEG classification performance.

First, random five-fold cross-validation was used for within-cohort benchmark classification. Windows were randomly partitioned without enforcing subject-level separation, so samples from the same participant could occur in different folds. This design estimates classification under sample-level mixing; it does not estimate generalisation to a previously unseen subject [14,15].

Second, leave-one-subject-out cross-validation (LOSO) was used to evaluate generalisation to unseen subjects. Let S=1,2,…,N denote the set of retained subjects. For each outer fold, one subject $s^{★}$ is held out for testing:

(13) $$S_{\mathrm{test}}=\left\{s^{★}\right\}.$$

The remaining subjects form the development set:

(14) $$S_{\mathrm{dev}}=S\setminus S_{\mathrm{test}}.$$

For the primary full-cohort GAF–PLV analysis, LOSO was performed on the 105 retained subjects, with one held-out test subject in each fold and a separate validation subject selected from the remaining subjects. This is the original full-cohort analysis and is kept distinct from the nested sensitivity analysis below.

Third, nested LOSO was used in the matched sensitivity analysis. In every outer fold, one subject was held out exclusively for final testing. Within the remaining subjects, five-fold GroupKFold model selection compared the three prespecified candidate configurations in Supplementary Table S3. This separation prevents the outer test subject from influencing hyperparameter selection and addresses model-selection bias [34,35]. Early stopping for deep models was applied only within inner-CV runs. After configuration selection, the final model was refitted on all outer-training subjects and tested once on the untouched outer subject. Because no separate validation subject was retained for that final refit, final-refit epoch optimisation was not independently tuned; this preserves outer-test independence but is a limitation for optimisation/generalisation and is acknowledged in the Discussion. No direction or magnitude of that effect is inferred from the present results.

For the GAF–PLV framework, window-level predictions were aggregated back to the trial level by majority vote:

(15) $${\hat{y}}_{s,r}=\mathrm{mode}\left\{{\hat{y}}_{s,r,1},{\hat{y}}_{s,r,2},\ldots ,{\hat{y}}_{s,r,10}\right\}.$$

This aggregation rule was used to obtain trial-level decisions from the ten non-overlapping windows extracted from each 4 s trial.

### 2.7. Performance Metrics

Performance was evaluated at the trial level. For binary classification, true positives (TP), true negatives (TN), false positives (FP), and false negatives (FN) denote correctly classified positive trials, correctly classified negative trials, incorrectly predicted positive trials, and incorrectly predicted negative trials, respectively. Accuracy is defined as

(16) $$\mathrm{Accuracy}=\frac{TP+TN}{TP+TN+FP+FN}.$$

Precision, recall, and F1-score are computed as

(17) $$\mathrm{Precision}=\frac{TP}{TP+FP},\;\mathrm{Recall}=\frac{TP}{TP+FN},$$

(18) $$F_{1}=\frac{2\cdot \mathrm{Precision}\cdot \mathrm{Recall}}{\mathrm{Precision}+\mathrm{Recall}}.$$

For multi-class evaluation, macro-averaged precision, recall, and F1-score were computed by averaging the corresponding class-wise metrics across classes. Cohen’s kappa is computed as

(19) $$\kappa =\frac{p_{o}-p_{e}}{1-p_{e}},$$

where $p_{o}$ is the observed agreement and $p_{e}$ is the expected agreement by chance. The area under the receiver operating characteristic curve (AUC) was computed from the predicted class scores; for multi-class classification, macro-averaged one-versus-rest AUC was reported.

### 2.8. Statistical Analysis

All statistical procedures are stated here before presentation of the results. For the primary 105-subject GAF–PLV LOSO analysis, held-out-subject folds are the statistical units. We report the mean, standard deviation, median, interquartile range, minimum, maximum, and a nonparametric subject-level bootstrap 95% confidence interval for the mean. Existing fold-level accuracy, Macro-F1, and kappa values were compared with their corresponding balanced-task reference values using two-sided one-sample *t* tests and two-sided Wilcoxon signed-rank tests. Cohen’s *d* denotes the one-sample standardised mean difference from the reference value. These tests describe the primary GAF–PLV fold distribution and are not used to establish superiority over the other classifiers.

For the matched N=30 analysis, the archived results available for the eight-classifier comparison are the aggregate accuracy, Macro-F1, and AUC values shown in Table 1 and Table 2; participant-level predictions or fold-level observations for every method–strategy combination are not available in a form that permits valid paired classifier inference. We therefore do not reconstruct standard deviations, confidence intervals, confusion matrices, or pairwise classifier *p* values from those aggregates, and classifier ordering is described as numerical ranking only.

To summarise the direction of validation-strategy changes without treating one classifier as the reference, we form 16 method–task pairs (8 classifiers × 2 classification tasks). For each comparison, Δ is defined as the first strategy minus the second strategy in percentage points. We report the number of positive differences, the mean and median Δ, and a two-sided exact sign test of whether positive and negative differences are equally likely. Because six strategy–metric sign tests are shown, Holm correction is applied across those six tests. These aggregate combinations share models and datasets and should not be treated as 16 independent participants; accordingly, the sign tests are an exploratory directional summary rather than a substitute for participant-level paired inference. Statistical significance, where used, is assessed at two-sided α=0.05 after the stated correction.

## 3. Results

We first report the predefined 30-subject matched sensitivity analysis, which examines whether changing the validation strategy changes aggregate performance and numerical ordering under otherwise matched conditions. We then report the retained 105-subject binary GAF–PLV LOSO analysis. These two levels answer different questions and are not pooled.

### 3.1. Protocol-Matched Performance on the Binary MI Task

Table 1 summarises the aggregate binary MI results across eight classifiers and three validation strategies. Within this predefined subset and common 0.4 s setting, the numerically highest-performing model under random five-fold cross-validation was ShallowConvNet (66.48% accuracy, 66.46% Macro-F1, 72.82% AUC). Under LOSO, DeepConvNet had the numerically highest accuracy (63.44%), Macro-F1 (63.22%), and AUC (68.66%). Under nested LOSO, ATCNet had the numerically highest accuracy (65.09%), Macro-F1 (65.00%), and AUC (71.12%). These are numerical rankings; the available aggregate baseline results do not support participant-level pairwise claims of classifier superiority.

The GAF–PLV-CNN accuracy was 56.98% under random five-fold cross-validation, 54.44% under LOSO, and 59.29% under nested LOSO. The sensitivity analysis is not designed to establish a definitive architecture leaderboard; rather, it shows that the numerical ordering changes when the validation strategy changes under a matched subset.

### 3.2. Protocol-Matched Performance on the Four-Class MI Task

Table 2 shows the corresponding four-class MI results. Aggregate accuracy was lower than in the binary task across all evaluated methods. Most aggregate accuracies remained above the nominal 25% chance level; no additional significance is inferred from that observation.

Within the predefined subset, ShallowConvNet was numerically highest under random five-fold cross-validation (45.19% accuracy, 45.15% Macro-F1, 71.75% AUC). Under LOSO, the numerical leader depended on the metric: EEGNet for accuracy (36.29%), DeepConvNet for Macro-F1 (34.87%), and GAF–PLV-CNN for AUC (63.76%). Under nested LOSO, EEG-Conformer had the highest numerical accuracy (37.82%), ATCNet the highest Macro-F1 (36.62%), and DeepConvNet the highest AUC (64.86%).

The GAF–PLV-CNN accuracy was 36.27% under random five-fold cross-validation, 31.59% under LOSO, and 31.94% under nested LOSO. Its aggregate Macro-F1 was lower under both subject-wise strategies than under random five-fold cross-validation, reinforcing the value of reporting class-balanced metrics alongside accuracy.

### 3.3. Effect of Validation Protocol on Model Ranking

**Figure 2:**
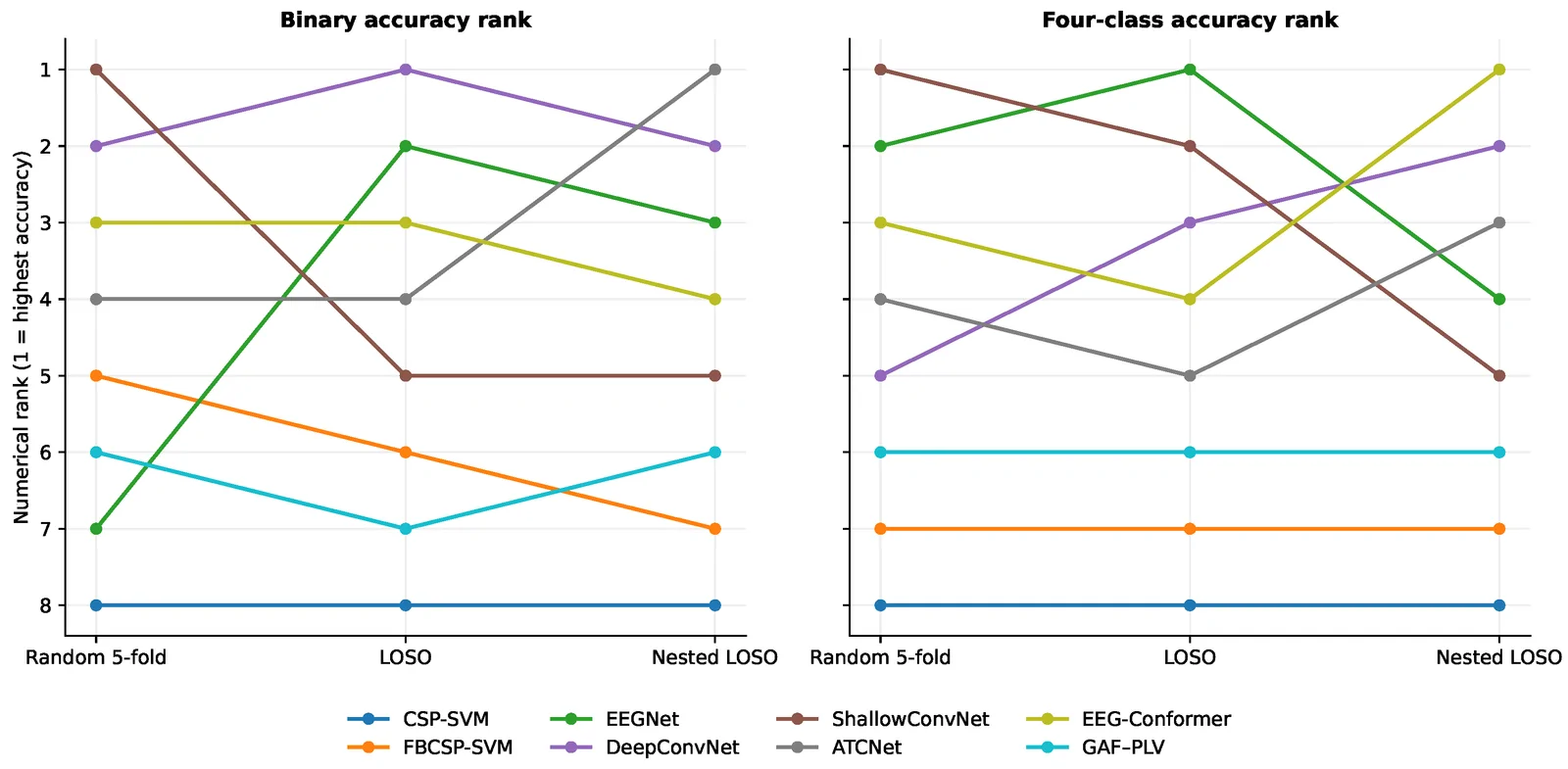
Change in numerical accuracy rank across validation strategies, calculated from the existing aggregate values in Table 1 and Table 2. Rank 1 denotes the highest aggregate accuracy within the predefined 30-subject, common 0.4 s analysis. The figure does not imply statistically significant differences between classifiers or full-cohort ranking stability.

The matched comparisons show that validation strategy affects both aggregate performance and numerical ordering within the predefined subset. Figure 2 displays the accuracy ranks derived directly from Table 1 and Table 2; it introduces no new model experiment. Binary rank 1 changes from ShallowConvNet (random five-fold) to DeepConvNet (LOSO) to ATCNet (nested LOSO), whereas four-class rank 1 changes from ShallowConvNet to EEGNet to EEG-Conformer.

Table 3 summarises the aggregate strategy differences across the 16 method–task pairs (8 methods × 2 tasks). Positive Δ means that the first strategy has the larger aggregate value. Random five-fold cross-validation was higher than LOSO for accuracy and Macro-F1 in 15 of 16 pairs, with mean differences of 3.03 and 4.77 percentage points, respectively. The corresponding exact sign tests remain below 0.05 after Holm correction. Because method–task pairs share data and model families, these tests are interpreted as directional summaries rather than participant-level evidence of a universal effect size.

The relationship between LOSO and nested LOSO is not monotonic. LOSO accuracy was higher in only 2 of 16 method–task pairs, yielding a mean LOSO-minus-nested difference of −1.74 percentage points. This negative difference does not define an intrinsic ordering of procedural difficulty. The strategies differ in model selection and final refitting, so their aggregate estimates can move in either direction while the outer nested test subject remains independent.

Taken together, the matched results show validation-strategy sensitivity in aggregate values and numerical rankings. They do not establish that the observed architecture ordering would be unchanged on all 105 retained subjects.

### 3.4. Full-Cohort GAF–PLV LOSO Analysis

The primary GAF–PLV framework was evaluated on the retained 105-subject binary cohort under subject-wise LOSO. Across the 105 held-out-subject folds, mean trial-level accuracy was 58.07% ± 8.27%, Macro-F1 was 53.48% ± 11.19%, macro precision was 60.97% ± 14.45%, and Cohen’s kappa was 0.1615 ± 0.1654. Median accuracy was 57.14% (IQR 52.38–64.29%; range 38.10–78.57%), demonstrating substantial between-subject variability.

Table 4 summarises the subject-wise descriptive and inferential statistics for these 105 held-out folds.

The fold-level analysis indicates that accuracy and Macro-F1 were above the 50% balanced-task reference in the existing one-sample tests, while the absolute kappa remained low. Kappa is closely linked to accuracy in this balanced binary setting and is therefore interpreted as a chance-corrected restatement of the same subject-level pattern rather than independent evidence of model superiority.

**Figure 3:**
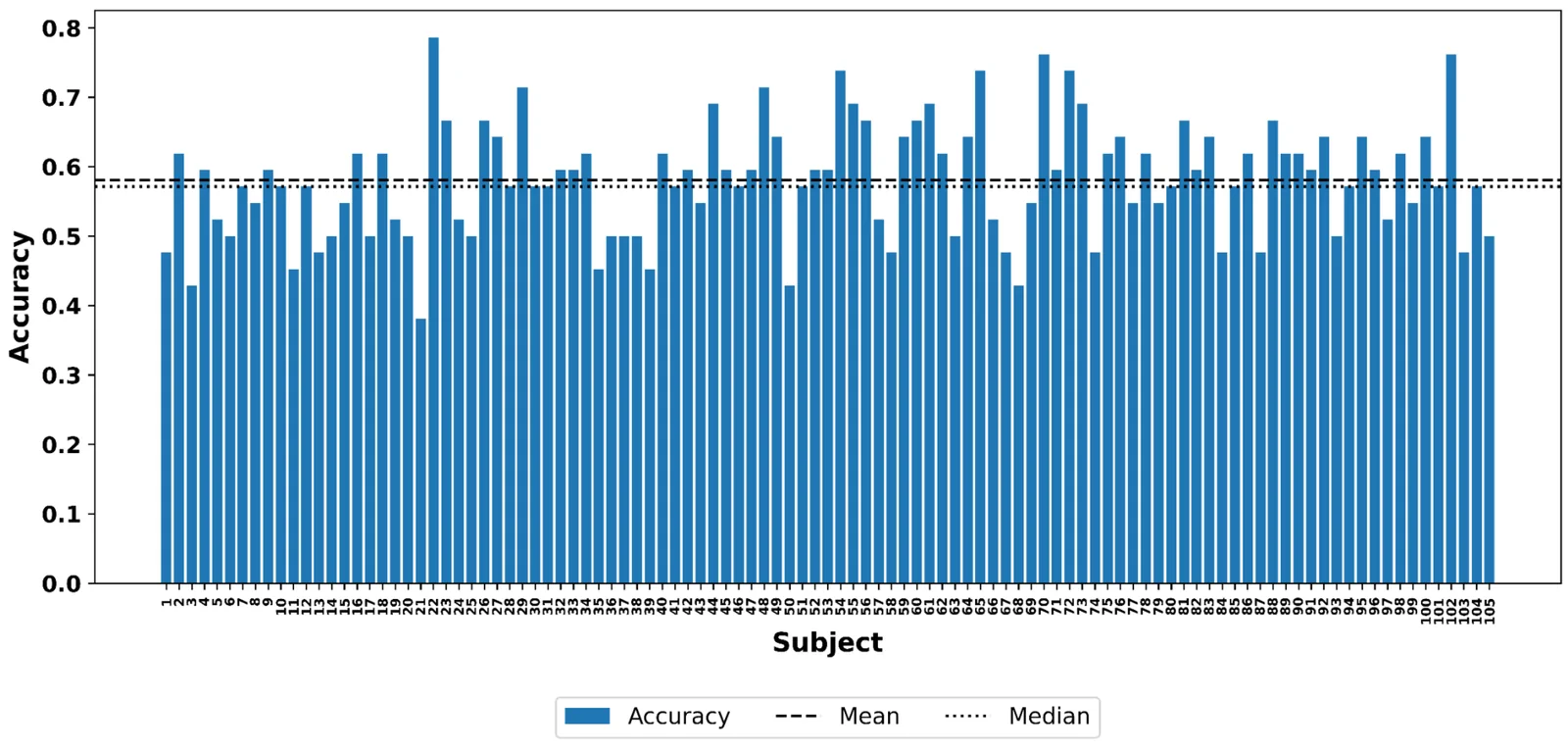
Subject-wise LOSO accuracy across all 105 retained folds for the primary GAF–PLV analysis. The dashed and dotted reference lines indicate the cohort mean and median, respectively.

The held-out subject profile in Figure 3 revealed substantial inter-subject variability. Subject 21 had the lowest fold accuracy (38.10%), whereas Subject 22 had the highest (78.57%); Subject 7 was at the cohort median (57.14%). Overall, 90 of 105 subjects (85.7%) reached at least 50% accuracy, 39 (37.1%) reached at least 60%, 8 (7.6%) reached at least 70%, and 3 (2.9%) exceeded 75%; 15 subjects (14.3%) were below 50%. These are descriptive subject-level results from the existing LOSO analysis.

**Figure 4:**
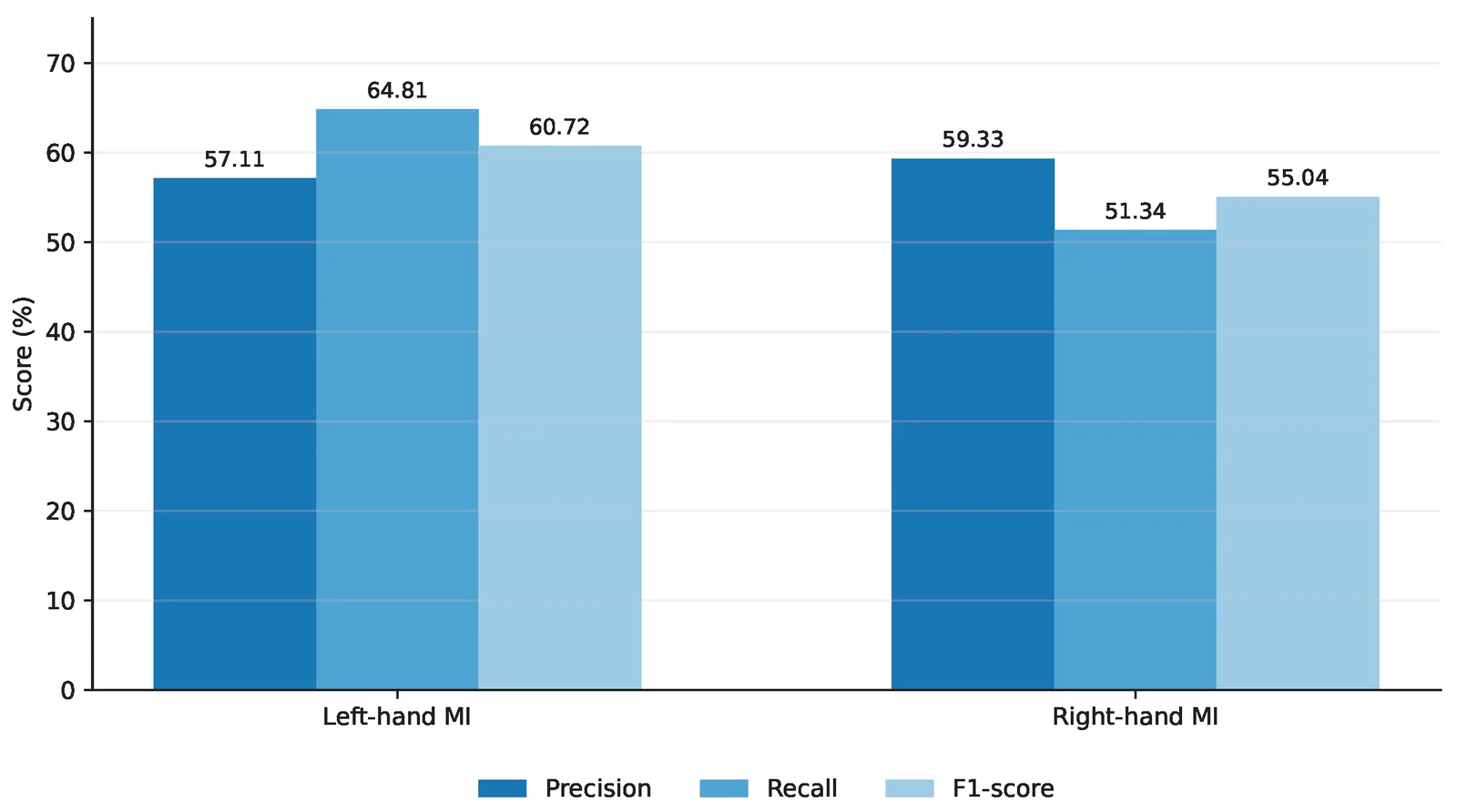
Corrected aggregate class-wise binary precision, recall, and F1-score for the retained 105-subject GAF–PLV LOSO analysis. Values are descriptive; fold-level class-wise observations were not available for paired inference.

The corrected aggregate class-wise values are shown in Figure 4. Left-hand MI precision, recall, and F1 were 57.11%, 64.81%, and 60.72%, respectively; right-hand MI values were 59.33%, 51.34%, and 55.04%. The submitted figure had inadvertently duplicated precision values in the recall bars; the revised figure uses the internally consistent values reported in the submitted text. Because archived fold-level class-wise observations or trial-level predictions are not available, the left–right difference is treated as descriptive only and no paired test, confidence interval, or mechanistic inference is reported.

The retained 105-subject analysis and the predefined 30-subject sensitivity analysis are therefore complementary rather than interchangeable: the former describes subject-wise transfer of the primary GAF–PLV framework, whereas the latter demonstrates validation-strategy sensitivity across multiple model families under matched conditions.

## 4. Discussion

### 4.1. Validation Target and Comparison with Published Work

The central result is that the scientific meaning of an MI-EEG score depends on the partition unit. In the predefined matched analysis, random five-fold cross-validation was higher than LOSO for accuracy and Macro-F1 in 15 of 16 method–task pairs. This direction is consistent with direct EEG evidence showing that segment-based holdout can retain subject-specific information and overestimate performance on previously unseen people [14,15]. Del Pup et al. likewise reported that partitioning choices materially affect supervised cross-subject EEG evaluation [16]. Our study extends this concern to a matched MI-EEG comparison in which the same subjects, temporal unit, tasks, and metrics were held constant while the validation strategy changed.

Headline comparison with published PhysioNet accuracies requires particular caution. PhysioNet MI-EEG studies differ in cohort size, task definition, preprocessing, temporal segmentation, and validation unit: for example, Lun et al. evaluated groups of 10–100 subjects using ten-fold cross-validation, Hwaidi and Chen evaluated 10 subjects, Fan et al. selected 20 PhysioNet subjects, and Aung et al. reported a 20-subject graph-based evaluation [22,24,25,26]. Other studies have used much larger retained cohorts but different pooled or subject-oriented partitions [19,20,21]. Consequently, a raw percentage from one paper cannot be interpreted as a controlled architecture comparison with the present 58.07% full-cohort GAF–PLV LOSO accuracy. The cohort, task, input duration, preprocessing, and subject separation must be matched before such an inference is defensible.

The distinction also matters for translational language. BCI-assisted rehabilitation has been tested in controlled clinical research [7], but an offline classifier evaluated on windows from already represented participants addresses a different question from a system intended to operate on a new user. Subject-wise validation is therefore especially relevant when claims concern calibration-free or previously unseen participants.

### 4.2. Why Numerical Model Rankings Can Change

The accuracy-rank changes in Figure 2 are descriptive but methodologically informative. ShallowConvNet, DeepConvNet, EEGNet, ATCNet, and EEG-Conformer do not impose the same inductive biases. Deep and shallow convolutional models differ in depth, temporal filtering, pooling, and nonlinearities [9]; EEGNet uses compact depthwise/separable convolutions and strong parameter sharing [8]; ATCNet combines attention with temporal convolution [10]; and EEG-Conformer combines convolutional patch embedding with transformer-based long-range dependency modelling [11]. CSP/FBCSP instead impose explicit spatial filtering before a linear classifier [28,33]. These differences can plausibly alter sensitivity to subject-specific covariance, temporal structure, capacity, and regularisation when the partition strategy changes.

However, the present experiments do not isolate those mechanisms. The model-specific explanations above are interpretations grounded in architecture, not experimentally demonstrated causes of the observed rank changes. They are further conditioned by the common 0.4 s input. In particular, attention- and transformer-based architectures originally evaluated on longer temporal segments may not express their intended temporal receptive fields optimally in 64 samples. A matched temporal duration improves control of the sensitivity analysis, but matching is not synonymous with architecture-specific optimality.

The GAF–PLV representation has a related interpretive boundary. GAF provides an image-like representation of within-channel temporal relations [30]; Spearman correlation then summarises monotonic similarity between channel-wise GAF patterns. This retains channel identities but does not encode physical electrode coordinates. PLV separately describes functional phase synchrony [31], which is also not the same as scalp geometry. Geometry-aware graph construction, electrode-coordinate priors, or topographic mappings could therefore be investigated in future work [12]. We no longer describe the GAF–Spearman descriptor as an explicit model of electrode topology.

### 4.3. Interpretation of the Retained 105-Subject GAF–PLV Analysis

The retained 105-subject binary GAF–PLV analysis addresses the study’s primary full-cohort question. Mean accuracy was 58.07% with an 8.27-percentage-point standard deviation, and the subject profile ranged from 38.10% to 78.57%. This spread is more informative for unseen-subject transfer than a single cohort mean because it shows that performance varies materially across held-out participants. The aggregate class-wise values also differ, particularly in recall, but the corrected class-wise figure is deliberately descriptive. Without archived fold-level class-wise observations or trial-level predictions, we cannot validly attach a paired confidence interval or test to the left–right difference, and we therefore do not infer a handedness, lateralisation, or other neurophysiological mechanism.

The full-cohort analysis was intentionally binary because it re-evaluates the previously developed GAF–PLV framework under subject-wise transfer for the left-versus-right MI problem. The four-class task was introduced in the separate sensitivity analysis to test whether validation-strategy effects persist under a harder label space and across multiple model families. A full-cohort four-class, eight-model nested experiment would answer an important additional question, but that experiment was not performed and no such result is implied here.

### 4.4. Limitations

Several limitations define the scope of the conclusions. First, the eight-model sensitivity analysis uses a predefined N=30 subset. Selection was outcome-independent, fixed-seed, and fully disclosed, which rules out performance-based subject selection, but subject identifiers are not biological strata and the subset is not claimed to be demographically, physiologically, or performance representative. The numerical architecture rankings therefore apply only to this predefined subset and do not establish ranking stability across all 105 retained subjects.

Second, all compared models used the same 0.4 s temporal unit. This controls input duration for the validation-strategy comparison but may disadvantage architectures whose original evaluations used longer temporal contexts [8,10,11]. The rankings should consequently be read as conditional on the common 0.4 s implementation rather than architecture-optimal rankings.

Third, the GAF–Spearman representation does not explicitly encode electrode geometry; PLV adds functional connectivity but not anatomical distance. Fourth, nested LOSO preserves outer-test independence, but after inner model selection the final model is refitted on all outer-training subjects without a separate validation subject. Early stopping is therefore confined to the inner runs, and final-refit epoch optimisation is not independently selected. We do not know from the present results whether that choice raises or lowers test performance.

Fifth, the public dataset release does not provide complete subject-level age, sex, and handedness metadata needed for stratified analyses. Sixth, the full-cohort analysis is limited to binary GAF–PLV LOSO; no 105-subject four-class or eight-model nested ranking experiment was performed. Seventh, all analyses use a single public dataset, so external-dataset generalisation was not tested. Finally, participant-level/fold-level outputs were not archived for every baseline–strategy combination, and trial-level predictions were not available for the retained full-cohort analysis; therefore new baseline confidence intervals, pairwise classifier tests, confusion matrices, a reconstructed empirical CDF, and paired class-wise inference cannot be generated without new experiments or unverifiable reconstruction.

The computational burden is supporting context rather than the scientific justification for the two analytical levels. The documented nested design requires 7680 fits/refits at N=30 and 26,880 at N=105, a 3.5-fold expansion. An author-provided pilot interval of 15:05–16:07 recorded 62 min for one representative deep-model training run on the current implementation. If that duration were held constant, the 1680 fits/refits required for one deep model at N=105 would project to approximately 1736 GPU-hours (72.3 continuous days). This is a projection for workload illustration, not a measured 105-subject runtime, and actual time varies by architecture and fold; it is therefore not extrapolated to the classical models.

Future work should test the stability of the multi-model rankings on the full retained cohort, include a full-cohort four-class analysis, compare architecture-specific input durations, incorporate explicit electrode geometry, and evaluate additional datasets. Those extensions would answer questions that the present validation-sensitivity study deliberately leaves open.

## 5. Conclusions

MI-EEG performance estimates and numerical model rankings changed when the validation strategy changed under matched conditions. The retained 105-subject binary GAF–PLV LOSO analysis yielded 58.07% ± 8.27% accuracy and substantial subject-to-subject variability, while the separate predefined 30-subject sensitivity analysis showed that the numerically highest-performing model was not invariant across random five-fold, LOSO, and nested LOSO evaluation. These findings support a precise reporting principle: within-cohort sample-level classification and generalisation to previously unseen subjects are different evaluation targets. The multi-model rankings are conditional on the predefined subset and common 0.4 s input setting; full-cohort multi-model and four-class ranking stability remains future work. Cohort definition, partition unit, validation design, and model-selection procedure should therefore be reported as core elements of MI-EEG evaluation.

## Figures and Tables

**Table 1:** Matched binary MI performance on the predefined 30-subject subset under the common 0.4 s input setting. Values are percentages. Acc denotes accuracy and F1 denotes Macro-F1. Rankings are descriptive because participant-level paired outputs are unavailable for all method–strategy combinations.

Method	Random 5-Fold			LOSO-CV			Nested LOSO-CV		
	Acc	F1	AUC	Acc	F1	AUC	Acc	F1	AUC
CSP-SVM	53.66	53.65	55.61	52.96	50.46	54.97	53.06	50.62	54.69
FBCSP-SVM	59.29	59.27	62.95	57.63	56.96	61.56	57.73	56.98	61.85
EEGNet	55.20	54.49	57.87	60.63	60.11	65.67	64.02	63.68	69.55
DeepConvNet	64.92	64.77	71.09	63.44	63.22	68.66	64.49	63.42	70.74
ShallowConvNet	66.48	66.46	72.82	58.94	58.00	63.33	60.08	57.76	65.56
ATCNet	63.47	63.44	69.32	59.06	58.65	63.40	65.09	65.00	71.12
EEG-Conformer	63.75	63.72	69.90	60.19	59.91	64.73	63.61	63.36	69.28
GAF–PLV-CNN	56.98	56.16	59.62	54.44	47.27	67.04	59.29	56.19	64.54

**Table 2:** Matched four-class MI performance on the predefined 30-subject subset under the common 0.4 s input setting. Values are percentages. Acc denotes accuracy and F1 denotes Macro-F1. Rankings are descriptive because participant-level paired outputs are unavailable for all method–strategy combinations.

Method	Random 5-Fold			LOSO-CV			Nested LOSO-CV		
	Acc	F1	AUC	Acc	F1	AUC	Acc	F1	AUC
CSP-SVM	30.48	30.35	57.05	28.65	25.71	55.39	29.06	26.32	55.38
FBCSP-SVM	34.03	34.00	60.59	30.72	28.20	57.54	30.67	27.96	–
EEGNet	39.92	39.94	66.46	36.29	34.37	62.62	36.59	35.05	63.24
DeepConvNet	37.65	37.01	64.22	35.79	34.87	62.10	37.77	34.66	64.86
ShallowConvNet	45.19	45.15	71.75	36.11	34.38	62.96	35.10	33.34	61.55
ATCNet	37.88	37.49	63.69	34.29	33.35	60.24	37.55	36.62	63.84
EEG-Conformer	39.45	39.18	65.93	35.32	33.79	61.69	37.82	36.61	63.61
GAF–PLV-CNN	36.27	36.00	64.44	31.59	25.53	63.76	31.94	27.46	59.16

**Table 3:** Aggregate validation-strategy differences across 16 method–task pairs (8 classifiers × 2 tasks). Positive Δ means that the first strategy produced the higher aggregate value. Exact sign-test *p* values are two-sided; $p_{\mathrm{Holm}}$ applies Holm correction across the six displayed tests.

Comparison	Metric	First Higher	Mean Δ (pp)	Median Δ (pp)	Exact *p*	$p_{\mathrm{Holm}}$
Random 5-fold − LOSO	Accuracy	15/16 (93.8%)	3.03	3.43	$5.19\times 10^{-4}$	0.00311
Random 5-fold − LOSO	Macro-F1	15/16 (93.8%)	4.77	4.72	$5.19\times 10^{-4}$	0.00311
Random 5-fold − Nested LOSO	Accuracy	12/16 (75.0%)	1.29	1.01	0.0768	0.1536
Random 5-fold − Nested LOSO	Macro-F1	13/16 (81.2%)	2.88	2.46	0.0213	0.0639
LOSO − Nested LOSO	Accuracy	2/16 (12.5%)	−1.74	−1.10	0.00418	0.0167
LOSO − Nested LOSO	Macro-F1	4/16 (25.0%)	−1.89	−0.65	0.0768	0.1536

**Table 4:** Subject-wise descriptive and inferential statistics across the 105 retained GAF–PLV LOSO folds. Accuracy and Macro-F1 are percentages; kappa is unitless. CI denotes the 95% bootstrap confidence interval of the mean.

Metric	Mean ± SD	Median	Q1–Q3	Min–Max	95% CI	Baseline	*t*(104)	Wilcoxon *p*	Cohen’s *d*
Accuracy (%)	58.07 ± 8.27	57.14	52.38–64.29	38.10–78.57	56.49–59.64	50	10.00 ($p<10^{-16}$)	$2.50\times 10^{-13}$	0.976
Macro-F1 (%)	53.48 ± 11.19	55.24	43.12–60.50	32.26–78.46	51.39–55.62	50	3.19 (p=0.0019)	$2.27\times 10^{-3}$	0.311
Cohen’s kappa	0.1615 ± 0.1654	0.1429	0.0476–0.2857	−0.2381–0.5714	0.1306–0.1927	0	10.00 ($p<10^{-16}$)	$5.84\times 10^{-14}$	0.976

## Data Availability

The EEG Motor Movement/Imagery dataset analysed in this study is publicly available from PhysioNet at https://www.physionet.org/content/eegmmidb/1.0.0/ (accessed on 1 January 2025). No new public dataset was created in this study.

## Supplementary Materials

The following supporting information can be downloaded at https://www.mdpi.com/article/10.3390/brainsci16080873/s1, Supplementary Table S1: Verified examples of reduced-cohort benchmarking in published PhysioNet MI-EEG studies. These examples establish precedent for reduced-cohort experiments but do not establish representativeness or full-cohort ranking stability; Supplementary Table S2: Subject identifiers used for the predefined matched sensitivity analysis; Supplementary Note S1: Hyperparameter settings and nested model selection (including Supplementary Table S3). Supplementary Table S3: Hyperparameter settings and modelselection strategy for the proposed model and protocol-matched baseline methods.
