# Supplementary material for: Evaluating Validation Strategies in Motor Imagery EEG: A Full-Cohort GAF–PLV Analysis and Matched Sensitivity Study
Source: Brain Sci. 2026 Aug 17;16(8):873. doi: 10.3390/brainsci16080873 (PMC13511235; doi:10.3390/brainsci16080873)
Supplement: Supplementary file 1 [file brainsci-16-00873-s001.zip › brainsci-4473534-supplementary.pdf]

Supplementary Materials: Evaluating Validation Strategies in Motor Imagery EEG: A Full-Cohort GAF–PLV Analysis and Matched Sensitivity Study

Table S1. Verified examples of reduced-cohort benchmarking in published PhysioNet MI-EEG studies. These examples establish precedent for reduced-cohort experiments but do not establish representativeness or full-cohort ranking stability.

Study	Year	PhysioNet subjects	Verified design note
Lun *et al.* [1]	2020	10, 20, 60, 100	CNN experiments were explicitly repeated at several group sizes.
Majoros and Oniga [2]	2022	10 and 20	The PhysioNet analysis reported reduced-cohort benchmarking as part of the study.
Hwaidi and Chen [3]	2022	10	The PhysioNet experiment reported aggregate results across 10 subjects.
Fan *et al.* [4]	2023	20	Twenty PhysioNet subjects were selected for evaluation alongside a second dataset.
Aung *et al.* [5]	2025	20	The published graph-based evaluation reported results across 20 PhysioNet subjects.

Table S2. Subject identifiers used for the predefined matched sensitivity analysis.

Selection group	Subject identifiers
Subjects selected from IDs 1–35	1, 3, 5, 11, 13, 15, 19, 22, 23, 35
Subjects selected from IDs 36–70	40, 43, 44, 55, 56, 57, 61, 63, 65, 70
Subjects selected from IDs 71–105	71, 73, 74, 77, 79, 84, 86, 94, 96, 102
Selection procedure	The 105 retained subjects were divided into three subject-ID ranges (1–35, 36–70, and 71–105). Using fixed random seed 2026, ten subjects were randomly sampled from each range before model fitting and without access to classifier performance. No performance-based inclusion or exclusion criterion was used. Subject-ID ranges are not biological strata and are not claimed to confer demographic, physiological, or performance representativeness.

Supplementary Note S1. Hyperparameter settings and nested model selection

This supplementary note reports the key implementation settings, hyperparameter search space, and nested model-selection procedure used for the proposed GAF–PLV–CNN and all protocol-matched baseline methods. The purpose of this note is to clarify how model comparison was controlled across different model families while preserving model-specific architectural requirements.

S1.1 Hyperparameter settings

The model-specific architecture, training, and hyperparameter-search settings are summarised in Table S3.

Table S3. Hyperparameter settings and model-selection strategy for the proposed model and protocol-matched baseline methods.

Model	Input and key architecture settings	Inner-CV training settings	Inner-CV search space	Input-specific adaptation
GAF–PLV–CNN	Paired GAF–Spearman and PLV images; 256 × 256 × 3 input; dual CNN branches followed by a merged dense layer	Adam optimiser; maximum 100 epochs; batch size 64 window representations; early-stopping patience 10	$(\text{lr}, \text{dropout}) \in \{(10^{-4}, 0.25), (3 \times 10^{-5}, 0.25), (10^{-5}, 0.50)\}$	Original image-based model; no input-length adaptation was required
EEGNet	Raw EEG windows; 64 channels × 64 samples; $F_1 = 8$, $D = 2$, $F_2 = 16$; temporal kernel length 32	Adam optimiser; maximum 100 epochs; batch size 64 windows; early-stopping patience 10	Same three $(\text{lr}, \text{dropout})$ configurations as GAF–PLV–CNN	Pooling configuration retained in a form compatible with 64-sample windows
DeepConvNet	Raw EEG windows; four convolutional blocks with 25, 50, 100, and 200 filters	Adam optimiser; maximum 100 epochs; batch size 64 windows; early-stopping patience 10	Same three $(\text{lr}, \text{dropout})$ configurations	Temporal convolutions used same padding to prevent collapse of the temporal dimension for 64-sample inputs
ShallowConvNet	Raw EEG windows; temporal convolution with 40 filters; spatial convolution; square activation; mean pooling; logarithmic activation	Adam optimiser; maximum 100 epochs; batch size 64 windows; early-stopping patience 10	Same three $(\text{lr}, \text{dropout})$ configurations	Pooling parameters were selected to maintain a valid temporal output for 64-sample windows
EEG-Conformer	Raw EEG windows; convolutional patch embedding; embedding size 40; six Transformer blocks; four attention heads	Adam optimiser; maximum 100 epochs; batch size 64 windows; early-stopping patience 10	Same three $(\text{lr}, \text{dropout})$ configurations	Patch-pooling stride set to 4 to preserve sufficient temporal tokens for 0.4-s windows
ATCNet	Raw EEG windows; EEG convolutional front end with $F_1 = 8$, $D = 2$, $F_2 = 16$; four-head attention; two TCN blocks with 32 filters	Adam optimiser; maximum 100 epochs; batch size 64 windows; early-stopping patience 10	Same three $(\text{lr}, \text{dropout})$ configurations	Temporal pooling set to 4 and 2; attention and TCN components were retained
CSP–SVM	Raw EEG windows; one-vs-rest CSP; four CSP components per class; linear SVM	Class-balanced SVM; standardised CSP features	$C \in \{0.1, 1, 10\}$; CSP components fixed at 4	Applied directly to the same 0.4-s EEG windows
FBCSP–SVM	Filter-bank EEG windows; six bands: 8–12, 12–16, 16–20, 20–24, 24–28, and 28–30 Hz; four CSP components per class; linear SVM	Class-balanced SVM; standardised FBCSP features	$C \in \{0.1, 1, 10\}$; CSP components fixed at 4	Filter bank restricted to the original 8–30 Hz preprocessing range; AUC calculated from SVM decision scores

S1.2 Nested model-selection protocol

For all models, the outer evaluation loop used leave-one-subject-out cross-validation. In each outer fold, the held-out participant was used only for final testing and was completely excluded from training, validation, preprocessing fitting, hyperparameter selection, early stopping, and model selection.

Within each outer-training set, five-fold GroupKFold cross-validation grouped by participant ID was used to evaluate the three prespecified candidate configurations listed in Table S3. For the deep-learning models, early stopping was applied only during these inner-CV training runs, with a maximum of 100 epochs, batch size 64 windows, and patience of 10 epochs. The candidate configuration with the highest mean inner-validation accuracy was selected for the corresponding outer fold.

After model selection, the final model for that outer fold was refitted using all available outer-training participants and the selected configuration, and was then evaluated once on the held-out outer-test participant. Because all outer-training participants were used for this final refit, no separate validation subject was retained at this stage; early stopping was therefore used only during inner-CV model-selection runs. The outer test subject remained untouched, so this procedure does not introduce outer-test leakage. However, the final-refit epoch is not independently optimised using a dedicated validation subject; this optimisation limitation is stated in the main Discussion. Any fitted preprocessing or feature-standardisation parameters were estimated only from the corresponding training data and then applied to validation or test data.

S1.3 Fairness of the protocol-matched comparison

The same 30-participant subset, 0.4-s EEG windows, outer LOSO folds, inner subject-grouped folds, random seed, and evaluation metrics were used across all compared models. Identical optimisation settings were applied to the deep-learning models wherever applicable. Model-specific hyperparameters were tuned only when they represented analogous core choices: learning rate and dropout for neural networks, and the SVM regularisation parameter C for CSP-based methods.

Because the evaluated methods belong to different model families, matching was defined by an identical subject subset, temporal duration, data partitioning, preprocessing,

metric reporting, and model-selection procedure rather than by forcing fundamentally different models to share irrelevant hyperparameters. The common 0.4-s duration is a controlled comparison condition, not evidence of architecture-specific optimality; the resulting numerical rankings are interpreted only under this matched input setting.

References

1. Lun, X.; Yu, Z.; Chen, T.; Wang, F.; Hou, Y. A simplified CNN classification method for MI-EEG via the electrode pairs signals. *Front. Hum. Neurosci.* **2020**, *14*, 338. https://doi.org/10.3389/fnhum.2020.00338.
2. Majoros, T.; Oniga, S. Overview of the EEG-based classification of motor imagery activities using machine learning methods and inference acceleration with FPGA-based cards. *Electronics* **2022**, *11*, 2293. https://doi.org/10.3390/electronics11152293.
3. Hwaidi, J.F.; Chen, T.M. Classification of motor imagery EEG signals based on deep autoencoder and convolutional neural network approach. *IEEE Access* **2022**, *10*, 48071–48081. https://doi.org/10.1109/ACCESS.2022.3171906.
4. Fan, C.; Yang, B.; Li, X.; Zan, P. Temporal-frequency-phase feature classification using 3D-convolutional neural networks for motor imagery and movement. *Front. Neurosci.* **2023**, *17*, 1250991. https://doi.org/10.3389/fnins.2023.1250991.
5. Aung, H.W.; Li, J.J.; Shi, B.; An, Y.; Su, S.W. EEG_GLT-Net: Optimising EEG graphs for real-time motor imagery signals classification. *Biomed. Signal Process. Control* **2025**, *104*, 107458. https://doi.org/10.1016/j.bspc.2024.107458.
